# Complete Genome Sequence of *Bacillus* sp. Strain TM2, Isolated from Ground *Tetragnatha* Spider Extract

**DOI:** 10.1128/mra.00130-22

**Published:** 2022-05-10

**Authors:** Masahiro Ito, Satomu Hasunuma

**Affiliations:** a Faculty of Life Sciences, Toyo University, Itakura-machi, Gunma, Japan; b Bio-Resilience Research Project, Toyo University, Itakura-machi, Gunma, Japan; c Bio-Nano Electronics Research Centre, Toyo University, Kawagoe, Saitama, Japan; University of Maryland School of Medicine

## Abstract

*Bacillus* sp. strain TM2, an aerobic Gram-positive bacterium, was isolated from a solution of ground Tetragnatha maxillosa spider in saline solution in Japan. Here, we report the complete genome sequence of this bacterium, which has a 3.67-Mbp genome containing 3,702 protein-coding sequences, 24 rRNA-coding sequences, and 81 tRNA-coding sequences.

## ANNOUNCEMENT

We report the whole-genome sequence of *Bacillus* sp. strain TM2, which was isolated from a solution of ground Tetragnatha maxillosa spider isolated in Japan (36.2231N, 139.6347E). The method for isolating strain TM2 from the spider was the same as that reported previously (1). TM2 is a high-concentration-cesium-resistant bacterium that can grow in the presence of 900 mM cesium chloride. This bacterium appeared to be most closely related to Bacillus altitudinis 41KF2b^T^, based on 16S rRNA gene sequence identity (2).

The method for preparing chromosomal DNA was the same as that reported previously (1). The exact same DNA extraction was used for both Oxford Nanopore Technologies (ONT) and Illumina libraries. For long-read sequencing, a DNA library with barcodes added to a sample using the native barcoding expansion kit (ONT, UK) was prepared using a ligation sequencing kit and sequenced with a GridION sequencer (ONT) and an R9.4.1 flow cell. In the library preparation, the genomic DNA was repaired and terminally modified without shearing of the genome, and then the adapters were ligated. The size selection process used AMPure XP beads (Beckman Coulter, USA) and the ligation sequencing kit to remove genomic DNA of less than 3,000 bp. Raw sequence data were base called using Guppy (v4.0.11+f1071ce) (3). The adapter sequences were removed using Porechop (v0.2.3) (4), and reads of 1,000 bases or less were removed using Filtlong (v0.2.0) (5), which yielded 163,106 high-quality paired-end reads. The average length of the long reads was 13,069 bp, and the total number of bases was 2,289,871,481 bp; the *N*_50_ value was 3,674,367 bp. For short-read sequencing, a DNA library was prepared using the MGIEasy FS DNA library preparation set (MGI Tech, China) according to the manufacturer’s instructions, and the library's quality was confirmed using a Fragment Analyzer system and the double-stranded DNA (dsDNA) 915 regent kit (Advanced Analytical Technologies Inc., USA). The reaction time for enzyme cleavage was 4 min, and an average fragment length of 442 bp was prepared. Circularized DNA was prepared using the prepared library and the MGIEasy circularization kit according to the manual. DNBSEQ 2 × 200-bp paired-end sequencing was performed using the DNBSEQ-G400 sequencer according to the manufacturer’s instructions. The adapter sequences were removed using Cutadapt (v2.7) (6). About 3.5 million read pairs (1.05 Gbp) were sampled from the sequence, from which the adapter sequences were removed using Seqkit (v0.11.0) (7); Sickle (v1.33) (8) was used to remove bases with quality scores of less than 20, and reads with fewer than 127 bases and their paired reads were discarded, yielding 6,265,860 high-quality paired-end reads. The average length of the short reads was 200 bp, and the total number of bases was 2,348,640,800 bp.

High-quality short-read and long-read sequence data were assembled under the default conditions of Unicycler (v0.4.7) (9), and the genome was circularized. The results of the coding graph assembled using Bandage (v0.8.1) (10) were confirmed, and the integrity of the assembled genomic data was confirmed using CheckM (v1.1.2) (11). The contamination estimated by CheckM1 was 0%. The coverage calculated from the total bases used for assembly was 1,260×. Default parameters were used for all software except where otherwise noted. The final chromosome sequence was 3,674,367 bp (G+C content, 41.4%), with an extrachromosomal plasmid of 7,196 bp (G+C content, 35.9%). Automatic annotation was performed using Annotated at DFAST (12), which predicted 3,702 coding sequences, 24 rRNA genes, and 81 tRNA genes.

### Data availability.

The GenBank accession number for the whole-genome sequence of *Bacillus* sp. strain TM2 is AP025262, and that for the extrachromosomal plasmid is AP025263. Raw sequencing data were deposited in the SRA under accession number DRA013015.
